# Nuclear ribonucleoprotein RALY downregulates foot-and-mouth disease virus replication but antagonized by viral 3C protease

**DOI:** 10.1128/spectrum.03658-23

**Published:** 2024-02-07

**Authors:** Jin'en Wu, Chao Sun, Junyong Guan, Sahibzada Waheed Abdullah, Xuefei Wang, Mei Ren, Lu Qiao, Shiqi Sun, Huichen Guo

**Affiliations:** 1 State Key Laboratory for Animal Disease Control and Prevention, College of Veterinary Medicine, Lanzhou University, Lanzhou Veterinary Research Institute, Chinese Academy of Agricultural Sciences, Lanzhou, China; 2 College of Veterinary Medicine, Gansu Agricultural University, Lanzhou, Gansu, China; 3 Division of Livestock Infectious Diseases, State Key Laboratory for Animal Disease Control and Prevention, Harbin Veterinary Research Institute, Chinese Academy of Agricultural Sciences, Harbin, China; 4 School of Animal Science, Yangtze University, Jingzhou, China; 5 Gansu Province Research Center for Basic Disciplines of Pathogen Biology, Lanzhou, China; Johns Hopkins Medicine, Baltimore, Maryland, USA

**Keywords:** foot-and-mouth disease virus (FMDV), RALY, internal ribosome entry site (IRES), virus-host interactions, 3C^pro^

## Abstract

The internal ribosome entry site (IRES) element constitutes a cis-acting RNA regulatory sequence that recruits the ribosomal initiation complex in a cap-independent manner, assisted by various RNA-binding proteins and IRES *trans*-acting factors. Foot-and-mouth disease virus (FMDV) contains a functional IRES element and takes advantage of this element to subvert host translation machinery. Our study identified a novel mechanism wherein RALY, a member of the heterogeneous nuclear ribonucleoproteins (hnRNP) family belonging to RNA-binding proteins, binds to the domain 3 of FMDV IRES via its RNA recognition motif residue. This interaction results in the downregulation of FMDV replication by inhibiting IRES-driven translation. Furthermore, our findings reveal that the inhibitory effect exerted by RALY on FMDV replication is not attributed to the FMDV IRES-mediated assembly of translation initiation complexes but rather to the impediment of 80S ribosome complex formation after binding with 40S ribosomes. Conversely, 3C^pro^ of FMDV counteracts RALY-mediated inhibition by the ubiquitin-proteasome pathway. Therefore, these results indicate that RALY, as a novel critical IRES-binding protein, inhibits FMDV replication by blocking the formation of 80S ribosome, providing a deeper understanding of how viruses recruit and manipulate host factors.

**IMPORTANCE:**

The translation of FMDV genomic RNA driven by IRES element is a crucial step for virus infections. Many host proteins are hijacked to regulate FMDV IRES-dependent translation, but the regulatory mechanism remains unknown. Here, we report for the first time that cellular RALY specifically interacts with the IRES of FMDV and negatively regulates viral replication by blocking 80S ribosome assembly on FMDV IRES. Conversely, RALY-mediated inhibition is antagonized by the viral 3C protease by the ubiquitin-proteasome pathway. These results would facilitate further understanding of virus-host interactions and translational control during viral infection.

## INTRODUCTION

Foot-and-mouth disease virus (FMDV), a single-stranded positive-sense RNA virus with an exclusively cytoplasmic life cycle, is the most economically important and highly contagious veterinary pathogen, seriously affecting livestock health and international trade of animals and related products (1, 2). As an important member of picornavirus, FMDV contains a long 5′-untranslated region (5′-UTR), a short 3′-untranslated region (3′-UTR), and an open reading frame (ORF) encoding a large polyprotein. This polyprotein undergoes subsequent cleavages by Lpro, 2A, and 3Cpro, leading to the generation of distinct protein products like Lpro, P1-2A, 2BC, and P3 (3). Subsequently, the precursor forms P1-2A, 2BC, and P3 undergoes additional cleavages to generate four mature structural proteins (VP4, VP2, VP3, and VP1) and eight non-structural proteins (Lpro, 2A, 2B, 2C, 3A, 3B, 3Cpro, and 3Dpol) (4, 5).

The genomic RNA of picornavirus differs from the cellular mRNA and does not depend on the canonical cap-dependent translation at the 5′ end. The 5′-UTR of FMDV genomes contain five functional elements: S fragment (S-frag), poly(C) tract, pseudoknot structures (PKs), *cis*-acting replicative element (Cre), and internal ribosome entry site (IRES) (6). IRES elements serve as the ribosome landing pads and play important roles in IRES-mediated translation by trapping shuttling IRES *trans*-acting factors (ITAFs) (7–9). Due to the absence of a complete life system for replication, the virus must hijack host proteins to form replication complexes (RCs), inhibit the synthesis of host proteins, rearrange cellular resources to facilitate viral replication, and disrupt the trafficking between the nucleus and the cytoplasm in infected cells for efficient viral IRES-driven translation and rapid adaption (10, 11). During ribosome complex recruitment, the core domain of IRES interacts with some canonical initiation factors and 40S sub-unit and then assembles an 80S ribosome to translate viral protein (12). Picornavirus IRES has been classified into five distinct types based on their use of ITAFs, eukaryotic initiation factors (eIFs), and the secondary structure of viral IRES sequence (13). The type II IRES of FMDV is one of the strongest IRESs and can quickly hijack the eIFs of host cells and initiate the translation of viral proteins (14). Canonical eIFs (eIF1A, eIF1, eIF2, eIF3, and eIF5), which are essential to initiate cap-dependent translation, are required to facilitate IRES-mediated translation. Although FMDV proteases Lpro and 3Cpro cleave eIF4G to strongly inhibit cellular capped mRNAs translation, eIF4G is required to reconstruct the translation initiation process by binding to IRES *in vitro* (14). Some RNA-binding proteins have also been investigated to act as ITAFs for viral translation. Polypyrimidine tract-binding protein (PTB) was the first ITAF to interact with FMDV IRES to promote FMDV translation, but it was subsequently confirmed to be cleaved during FMDV infection (15, 16). After the discovery of PTB function in regulating FMDV IRES-mediated translation, several ITAFs such as ITAF45, Sam68, G3BP1, hnRNP K, DDX23, DDX3, and RPL13 were also found to regulate FMDV life cycles by affecting IRES activity (4, 17–22). Interestingly, the latest research shows that hnRNP L inhibits the growth of FMDV not by regulating IRES-mediated translation but by affecting viral RNA synthesis via IRES (23). These results indicated that these ITAFs have diverse functions in FMDV replication.

Given the important function of FMDV IRES in interacting with host factors, our previous studies used FMDV IRES as bait through an RNA pulldown assay, and proteomics approaches identified several host proteins interacting with FMDV IRES (4). Among those proteins, the RNA-binding protein RALY (also known as hnRNPCL2) belongs to the hnRNP family and contains an RNA recognition motif (RRM), making RALY with RNA-binding properties (24). Previous studies have shown that RALY functions in mRNA splicing and metabolism(25) and adenovirus late RNA splicing (26). A recent study also found that RALY significantly promotes the degradation of the PEDV nucleocapsid (N) protein to inhibit viral replication (27). Besides its role in viral infection, RALY also promotes cancer in multisystem tumors, promoting the proliferation of breast cancer, and promotes cancer in multisystem tumors, promoting the proliferation of breast, hepatocellular carcinoma, and cervical cancer cells (28). These data suggested that RALY is a multifunctional RNA-binding protein involved in many biological processes.

Our study focused on the interaction between RALY and IRES to determine the effects on FMDV replication. These results indicated that RALY interacts with domain 3 (D3) of FMDV IRES through its RNA-binding residue RRM to inhibit IRES-driven translation by blocking 80S ribosome assembly on FMDV IRES. Conversely, RALY-mediated inhibition was counteracted by FMDV 3C^pro^ via the ubiquitin-proteasome pathway. Thus, our results reveal a new mechanism of RALY in inhibiting viral replication and provide novel insights for developing broad anti-viral interventions.

## MATERIALS AND METHODS

### Cells, viruses, and plasmids

Baby hamster kidney cells [BHK-21, American Type Culture Collection (ATCC) CCL-10], porcine kidney (PK-15, ATCC CCL-33), and BSR-T7 cells, which stably express T7 RNA polymerase, were cultured in Dulbecco’s modified Eagle’s medium (Gibco, CA, USA) containing 10% fetal bovine serum (FBS, Gibco), 100-U/mL penicillin, and 100-µg/mL streptomycin. FMDV strain O/China/99 (GenBank accession no. AF506822.2) is stored by the OIE/National Foot-and-Mouth Disease Reference Laboratory (Lanzhou, China). FMDV was grown in BHK-21 cells, and virus titer was determined with a TCID_50_ assay.

### Antibodies and reagents

The anti-eIF4G, anti-ribosomal protein S5 (RPS5), anti-RPLP0, anti-eIF2a, anti-eIF3A, anti-eIF3e, anti-eIF4A, anti-eIF5B, and anti-RALY monoclonal antibodies were purchased from Abcam (Cambridge, MA, USA). Anti-FLAG monoclonal antibody was purchased from Proteintech (Chicago, IL, USA). Anti-β-actin monoclonal antibody was purchased from Kangwei Century (Jiangsu, China). Anti-HA monoclonal antibody, the protein synthesis inhibitor cycloheximide (CHX), and secondary antibodies conjugated with horseradish peroxidase (HRP) were purchased from Sigma-Aldrich (St. Louis, MO, USA). Polyclonal pig antiserum directed against FMDV and polyclonal rabbit antiserum against SVA (Senecavirus A) and EMCV (Encephalomyocarditis virus) were generated and stored in our laboratory. Transfection reagents Lipofectamine LTX for transfection recombinant plasmids and Lipofectamine RNAiMax Reagent for small interfering RNA (siRNA) transfection were purchased from Invitrogen (CA, USA). The RNA extraction reagent TRIzol was purchased from Invitrogen.

### Plasmid construction

The total RNA of BHK-21 cells was extracted and used as the template for amplifying the cDNA of RALY. The cDNA fragments of the pCMV-N-Flag vector and RALY were digested by *Eco*RI and *Xho*I endonuclease, respectively, and the cDNA fragments of RALY were inserted into pCMV-N-FLAG skeleton vector to obtain FLAG-RALY recombinant plasmids, respectively. The pET28a vector (Clontech, USA) and RALY cDNA were synthesized by Sangon Biotech (Shanghai). The cDNAs of FMDV non-structural proteins L^pro^, 2B, 2C, 3A, 3B, 3C^pro^, and 3D^pol^ were cloned from the genome of FMDV strain O/BY/CHA/2010 and then inserted into the pCMV-N-Flag vector. Several residues of Flag-3C^pro^ were mutated with a site-directed mutagenesis kit (Agilent Technologies, CA, USA) to generate Flag-3C^pro^ mutants H46Y, H84N, C163G, and H205R. psiCHECK-FMDV was constructed as previously described (4). All the recombinant plasmids were verified with DNA sequencing.

### *In vitro* transcription and synthesis of biotinylated RNA

The DNA templates for *in vitro* transcription were produced and linearized as previously described (14). Briefly, the viral cDNAs corresponding to 5′-UTR, the S-frag, the Cre, the IRES, and 3′-UTR of FMDV genome were amplified and inserted into the pcDNA3.1 vector and linearized by BamH I. FMDV RNA fragments were then generated by RiboMAX large-scale RNA production systems-T7 (Promega). Biotinylated RNAs were produced with a Pierce RNA 3′ End Desthiobiotinylation Kit (Thermo Scientific). The RNA fragments were then purified with TRIzol and dissolved in an appropriate buffer.

### RNA immunoprecipitation and reverse transcription (RT)-PCR

BHK-21 cells were infected with FMDV at a multiplicity of infection (MOI) of 1. At 5 h post-infection (hpi), the cells were treated with radioimmunoprecipitation assay (RIPA) buffer (Beyotime Biotechnology) containing a protease inhibitor and RNase inhibitor, and the supernatant of the cell lysate was collected. Protein G beads were mixed with the cell lysate, incubated on ice for 1 h, and then centrifuged at 3,000 × *g* for 10 min at 4°C to remove the complexes non-specifically bound to the protein G beads. Then 8 µL of anti-RALY or immunoglobulin G (IgG) antibody was added to the cell lysate, or no antibody was added as the negative control. The samples were rotated overnight at 4°C. The pre-treated protein G beads were added and incubated for 2–4 h at 4°C. The mixture of immune complexes was collected by centrifugation at 3,000 × *g* for 5 min at 4°C and washed three times with lysis buffer. The complexes were then resuspended and precipitated in 400 µL of buffer [100-mM Tris-HCl (pH 8.0), 12.5-mM EDTA, 150-mM NaCl, and 1% SDS] containing albumin K and incubated for 30 min at 37°C. The total RNA was extracted with TRIzol Reagent (Invitrogen), and an RT-PCR analysis, with the One Step RT-PCR Kit (Takara), was performed to detect the FMDV IRES, ORF, and 3′-UTR, and the RPS16 and GAPDH gene fragments.

### RNA interference

The cell culture medium was discarded when cells were grown to 70% confluence, and a cell maintenance solution containing 2% FBS was added. The cells were transfected with siRNAs with Liposome RNAiMAX Reagent (Invitrogen) and incubated for 36–48 h. The siRNAs targeting the candidate genes (siRALY-sense 5′-GCCUUUGUCCAGUAUGCCATT-3′, antisense 5′-ACGUGACACGUUCGGAGAAT-3′) and negative control (siNC-sense 5′-UGGCAUACUGGACAAAGGCTT-3′, antisense 5′-ACGUGACACGUUCGGAGAAT-3′) were synthesized by GenePharma (Shanghai, China).

### Immunoprecipitation assay

BHK-21 cells were co-transfected with the appropriate recombinant plasmids, and after incubation for 24 h, the cells were collected and lysed with RIPA buffer containing a protease inhibitor. Each cell lysate was centrifuged at 15,000 × *g* for 20 min at 4°C, and the supernatant was collected. An aliquot of the supernatant (50–100 µL) was reserved as the input sample. The appropriate antibody was added to the remaining supernatant, which was rotated at 4°C. The protein samples containing the incubated antibodies were added to pre-treated protein G beads and incubated at 4°C for 2–4 h. The antibody-bead mixture was centrifuged at 3,000 × *g* for 30 s at 4°C, and the supernatant was discarded. After the beads were washed two to three times with lysis buffer containing a protease inhibitor and reductant, 50 µL of 1× SDS loading buffer was added, and the samples were boiled in a metal bath for 5–10 min before analysis with Western blotting.

### Luciferase reporter assays

After BHK-21 cells were knocked down or overexpressed, the cells were transfected with the corresponding bicistronic reporter plasmid, psiCHECK or psiCHECK-FMDV, respectively. At 24 h post-transfection, the cells were lysed by the special cell lysate buffer for dual luciferase reporter assay, and the signal intensities of *Firefly* luciferase (*Fluc*) and *Renilla* luciferase (*RLuc*) were detected with the Dual-Luciferase Reporter Assay Kit (Promega) according to the manufacturer’s instructions. Monocistronic luciferase activity was measured in the rabbit reticulocyte *in vitro* translation system [rabbit reticulocyte lysate (RRL), Promega] as previously described (29). The template with pGL3-FMDV IRES-*FLuc* gene for *in vitro* translation was synthesized by Azenta Life Sciences (Suzhou, China). RALY (50 pmol) or a non-specific control protein, BSA, and 0.5 µg of template RNA *in vitro* transcript were added to 200 µL of the reaction system and incubated at 30°C for 40 min, and luciferase activity was immediately determined with Steady-Glo (Promega).

### Quantitative real-time PCR

BHK-21 cells were transfected with Flag-RALY or the empty FLAG vector and incubated for 24 h, or BHK-21 cells were transfected with RALY siRNA or NC siRNA and incubated for 48 h and then infected with FMDV (MOI = 1). At 1, 3, 5, 7, and 9 hpi, the total RNA in the cell lysates was extracted with TRIzol Reagent and reverse transcribed to cDNA. Quantitative PCR (qPCR) was then used to detect the expression levels of the target genes. The primer sequences are shown in Table 1.

**TABLE 1:** Primers used in this study

No.	Primer name	Sequence (5′−3′)
1	FMDV-Fwd	CAAACCTGTGATGGCTTCGA
2	FMDV-Rev	CCGGTACTCGTCAGGTCCA
3	hGAPDH-Fwd	GTCCATGCCATCACTGCCACCCAG
4	hGAPDH-Rev	GCTGTTGAAGTCACAGGACACAAC
5	IRES-Fwd	CACAGGTTCCCACAACCGACAC
6	IRES-Rev	GCAGTGATAGTTAAGGAAAGGC
7	3D-Fwd	GTTGCTAGTGATTATGACTTGGAC
8	3′UTR-Rev	CTTACGGCGTCGCTCGCCTCAGAG
9	hRPS16-Fwd	TCGCAGCCATGCCGTCCAAGGGT
10	hRPS16-Rev	TCATTAAGATGGGCTCATCGGT
11	GAPDH-Fwd	TCCATGCCATCACGGCCACCCAG
12	GAPDH-Rev	ACTC TTGAAGTCGCAGGAGACAAC

### Sucrose gradient sedimentation

Mock- or FMDV-infected cells were incubated with 0.1-mg/mL CHX for 15 min at 37°C to arrest the ribosome. At 4 hpi, cells were collected and lysed using polysomal extraction buffer [20-mM Tris-HCl (pH 7.5), 5-mM MgCl_2_, 100-mM KCl, 1% Triton X-100, 0.1-mg/mL CHX, protease inhibitor cocktail (EDTA-free), and 50 U/mL RNase inhibitor]. The lysates from FMDV-infected cells were treated by RNase I or EDTA on ice for about 40 min. The cell lysates were centrifuged to remove cellular debris at 12,000 × *g* for 10 min at 4°C, and the supernatants were fractionated on a linear 5%–50% sucrose gradient by centrifugation at 36,000 rpm at 4°C for 3 h in an SW40 Ti rotor (Beckman). Fractions (~500 µL) were collected from the top of the gradient, and the optical density was measured at a wavelength of 280 nm. The proteins in the fractions were analyzed by Western blotting.

### Western and dot blotting

After SDS-PAGE, the proteins were transferred to polyvinylidene fluoride (PVDF) membranes and blocked with 1× Tris-buffered saline with Tween 20 (TBST) containing 5% skimmed milk for 1 h, or the different fraction samples from the RALY and BSA groups were added to each square with 7 µL per square. After natural drying, the PVDF membrane was blocked with 5% skimmed milk. The corresponding primary antibody (diluted 1:1,000) was then incubated with the membrane overnight at 4°C. The membrane was washed four times with 1× TBST on a shaker for 5 min each time. The membrane was then incubated with the corresponding HRP-labeled secondary antibody (diluted 1:4,000). The membrane was washed four times with 1× TBST on a shaker for 5 min each time. Finally, ECL chromogenic solution (Sigma-Aldrich) was added then exposed.

### TCID_50_ assay

Various proteins were knocked down or overexpressed in BHK-21 cells, which were then infected with FMDV (MOI = 1). The infected cells (including the cell supernatant) were collected at different time points and freeze-thawed three times. The collected cell fluid was then serially diluted and added to a 96-well plate with eight wells per sample at 100 µL per well. Then 100 µL of BHK-21 cell suspension (1.5 × 10^6^ /mL) was added to each well, and the cells were incubated at 37°C in a 5% CO_2_ incubator for about 70 h to determine the number of cytopathic effects, and the TCID_50_ was calculated with the Reed-Muench method. Each datum is the mean result of three independent experiments.

### Statistical analysis

All data presented in this paper are expressed as the means ± standard deviations. Statistical significance was determined with Student’s *t*-test in GraphPad Prism software version 9 (GraphPad, La Jolla, CA, USA) and assessed based on the *P* value: **P＜0.01.

## RESULTS

### FMDV infection causes the decrease of RALY protein

**Fig 1:**
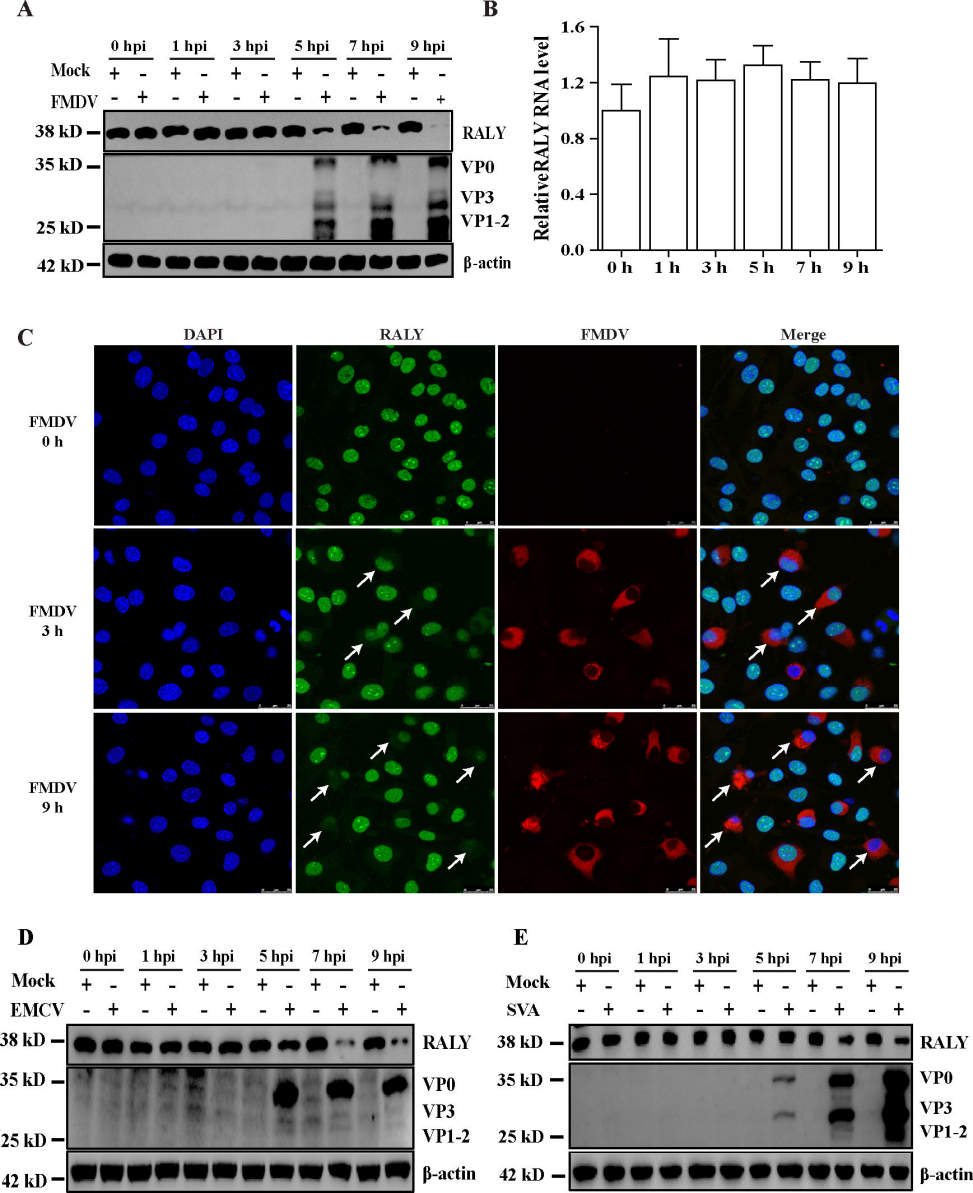
FMDV infection causes the decrease of RALY protein. (**A**) The RALY and FMDV structure proteins were detected by Western blot at indicated time points. (**B**) BHK-21 cells were infected with FMDV, and total RNA was extracted to examine the effect of FMDV on the transcription level of RALY mRNA. (**C**) BHK-21 cells were mock or FMDV infected and then fixed at 4 or 6 hpi. The cells were permeabilized and then probed with indirect immunofluorescence for FMDV (red) and RALY (green). Nuclei are indicated by DAPI staining (blue). Cells were observed by confocal microscopy. (**D and E**) The RALY and FMDV structure proteins were detected in EMCV-infected BHK-21 and SVA-infected IBRS-2 cells by Western blot at indicated time points.

To assess the RALY protein and mRNA level in FMDV-infected cells, BHK-21 cells were infected with type O FMDV (MOI of 1) and harvested at 0, 1, 3, 5, 7, and 9 hpi. Western blotting results indicated that RALY was decreased at 5, 7, and 9 hpi (Fig. 1A). Next, the lysates of BHK-21 cells infected with type O FMDV were collected using TRIzol Reagent for RNA extraction. The qPCR results showed that the transcript levels of RALY mRNA did not change significantly at indicated time points (Fig. 1B). These results indicated that FMDV infection causes a decrease in RALY protein, not RNA transcript.

RALY is an RNA-binding protein mainly located in uninfected cells’ nuclei. However, FMDV is a fast-replicating RNA virus in the cytoplasm of a cell. To explore whether RALY was redistributed from the nucleus to the cytoplasm during FMDV infection, immunofluorescence assay was performed to visualize this phenomenon. As expected, RALY showed nucleocytoplasmic relocalization at 3 and 9 hpi, but most of the RALY was degraded in the cytoplasm at 9 hpi (Fig. 1C). Meanwhile, we also examined the effect of EMCV, SVA, and other picornaviruses, on RALY protein. As shown in Fig. 1D and E, RALY was significantly reduced in EMCV-infected BHK-21 and SVA-infected IBRS-2 cells, respectively, indicating that the reduction of RALY protein may be a general phenomenon in picornaviruses.

### The effect of RALY on FMDV replication in infected cells

**Fig 2:**
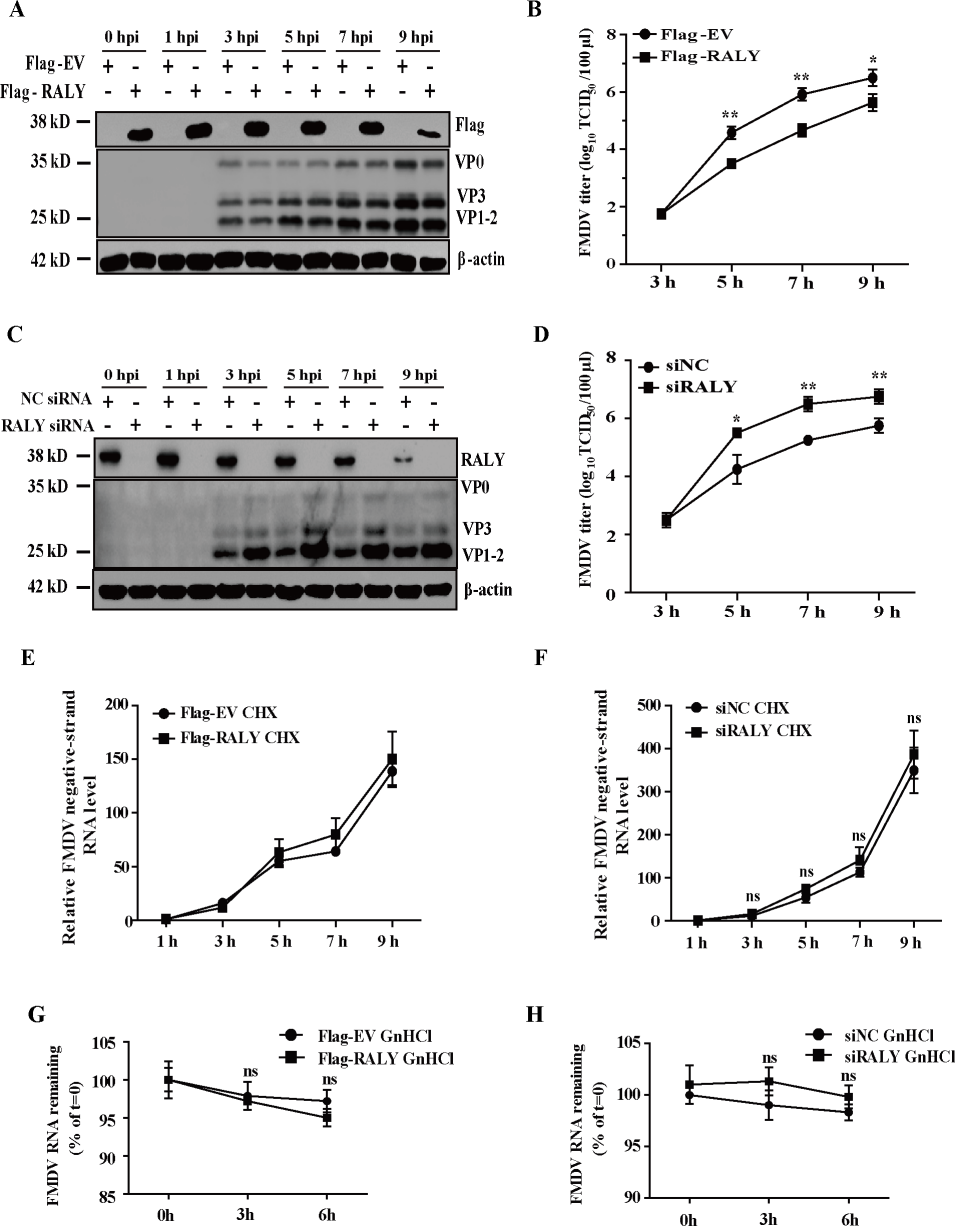
The effect of RALY on FMDV replication in infected cells (**A**) BHK-21 cells were transfected with Flag-RALY and empty vector (EV) for 24 h, followed by FMDV infection. Cell lysates were harvested at directed time points and subjected to Western blot analysis. (**B**) Viral progeny yields were examined based on TCID_50_ analysis. (**C**) BHK-21 cells were transfected with siRALY or siNC. After 36 h, the cells were infected by FMDV. Cell lysates were harvested at directed time points and subjected to Western blot analysis. (**D**) Viral progeny yields were examined based on TCID_50_ analysis. (**E and F**) The RALY-overexpressing or RALY-depleting BHK-21 cells were infected with FMDV, and CHX (100 mg/mL) was added to the culture medium at 3 hpi. Total RNA was extracted at indicated time points, and the negative-strand RNA was determined by RT-qPCR. (**G and H**) The degradation of FMDV mRNA was evaluated in RALY-overexpressing or RALY-depleting BHK-21 cells treated with the guanidine hydrochloride (GnHCl) at 5 h after FMDV infection. Total RNA was extracted at directed time points, and the levels of total viral RNA remaining were determined by RT-qPCR. Data were analyzed with Student’s *t*-test in GraphPad Prism software version 9 (GraphPad, La Jolla, CA, USA) and assessed based on the *P* values. *P＜0.05, **P ＜0.01. ns, not significant.

To further confirm the effect of RALY on FMDV replication in infected cells, RALY was firstly overexpressed or knocked down by transfecting hamster-derived pCMV-RALY-Flag or specific siRNA targeting RALY mRNA into BHK-21 cells, respectively. As shown in Fig. 2A and C, RALY was ectopically expressed and knocked down in FMDV-infected cells compared with EV-Flag and siNC-transfected groups. The FMDV protein production and viral progeny yields were analyzed to assess the impact of RALY on FMDV replication. The ectopic expression of RALY reduced the production of viral protein and progeny virus yields at 3, 5, 7, and 9 hpi, respectively (Fig. 2A and B). In contrast, the viral protein and titer were increased in RALY-depleted BHK-21 cells (Fig. 2C and D). These results suggest that RALY plays a negative role during FMDV replication.

To validate whether it affects the synthesis and stability of viral mRNA, the protein synthesis inhibitor CHX was used to monitor the viral negative-strand RNA synthesis. As shown in Fig. 2E and F, when viral protein translation was blocked at 3 hpi, there was no significant difference in viral negative-strand RNA synthesis whether RALY was ectopically expressed or depleted, demonstrating that RALY does not directly affect viral RNA synthesis. Since RALY can regulate the stability of some mRNA (30), we further explore the fate of viral RNA using the guanidine hydrochloride, an inhibitor of FMDV RNA synthesis in FMDV-infected BHK-21 cells. As shown in Fig. 2G and H, no significant differences were observed in RALY-overexpressing or RALY-silencing BHK-21 cells, revealing that RALY is not required for maintaining viral RNA stability.

### RALY specifically interacts with FMDV IRES

**Fig 3:**
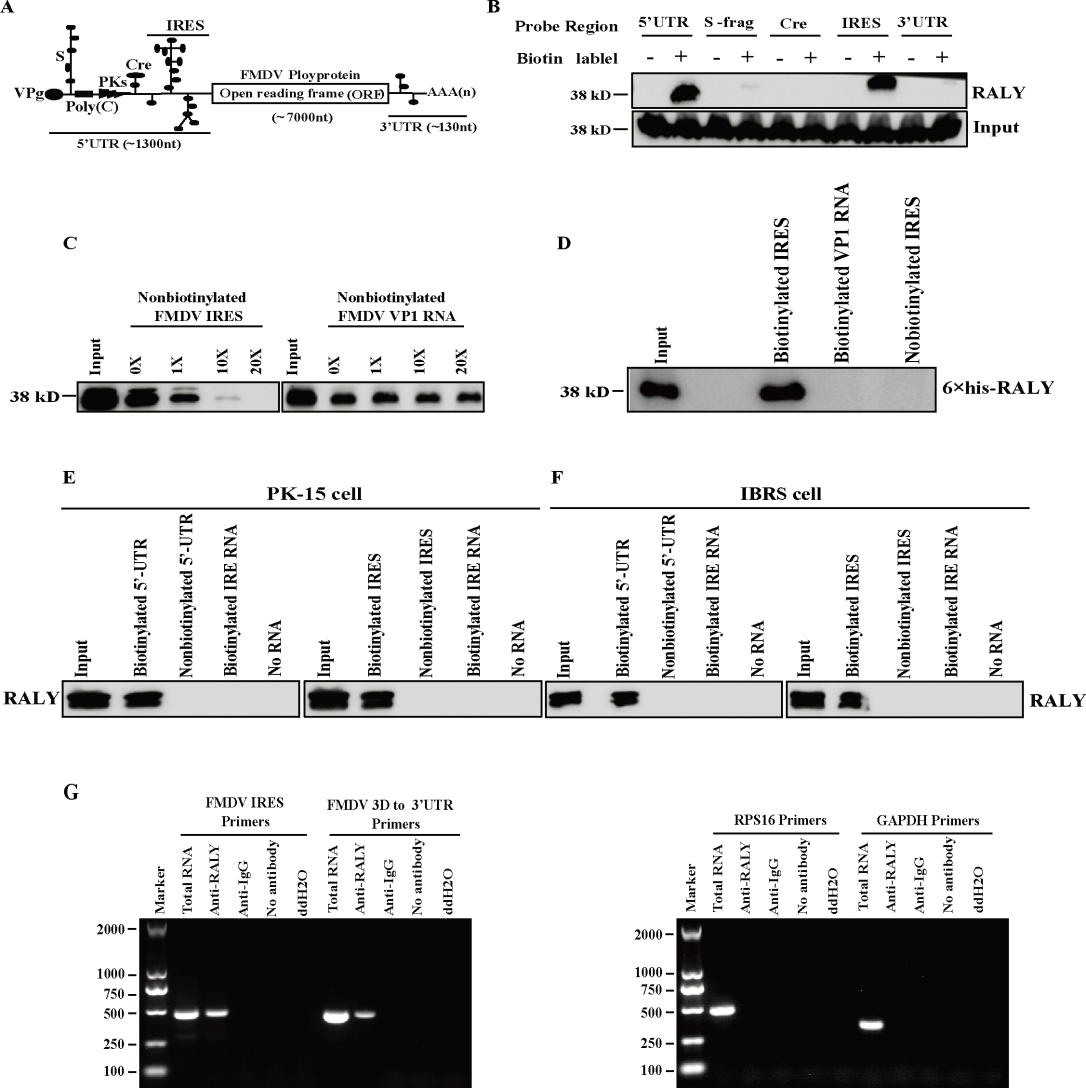
The interaction of RALY and FMDV IRES. (**A**) Schematic diagram of the FMDV genome. (**B**) The cell lysates of BHK-21 were incubated with the biotin-labeled 5′-UTR, S-frag, Cre, IRES, and 3′-UTR of FMDV, respectively. Non-biotinylated RNA probes were utilized as negative controls. These streptavidin beads were washed following the manufacturer’s instructions and subjected to immunoblot analysis with anti-RALY monoclonal antibody. (**C**) The different amounts of non-biotinylated FMDV IRES and VP1 RNA were added to compete with the biotinylated FMDV IRES that interacted with RALY. (**D**) The biotinylated FMDV IRES, VP1 RNA, and the purified recombinant 6 × His RALY expressed in *E. coli* were performed in RNA-pulldown assay, and non-biotinylated RNA probes were used as a negative control. (**E and F**) The cell lysates of PK-15 and IBRS-2 were incubated with the biotin-labeled 5′-UTR or IRES. The protein complexes were pulled down and subjected to Western blot analysis. (**G**) The FMDV-infected BHK-21 cells were lysed and incubated with an anti-RALY antibody for RNA immunoprecipitation. Negative controls included anti-IgG, no antibody, and ddH_2_O. RNA was extracted and amplified by RT-PCR using primers directed against FMDV IRES, 3D to 3′-UTR, RPS16, and GAPDH.

Our previous LC-ESI-MS/MS study showed that RALY associates with the FMDV 5′-UTR or IRES (4). We performed an RNA-pulldown assay to further determine this mechanism of IRES-associated RALY in FMDV translation and replication. BHK-21 cell extracts were incubated with biotinylated-RNA probes, which include the functional elements in the 5′-UTR, S frag, Cre, IRES, and 3′-UTR of FMDV genome (Fig. 3A). The results revealed that RALY was pulled down by the biotinylated 5′-UTR or IRES but not with S-frag, Cre, and 3′-UTR (Fig. 3B). Meanwhile, a competition experiment was performed by the RNA affinity assay to assess whether different amounts of non-biotinylated IRES influenced the interaction between RALY and the biotinylated FMDV IRES. As shown in Fig. 3C, the interaction was outcompeted by non-biotin-FMDV IRES rather than FMDV VP1 RNA, indicating that RALY specifically interacts with FMDV IRES. To confirm whether RALY directly binds to the FMDV IRES, we performed the RNA-pulldown assay using the biotinylated FMDV IRES, VP1 RNA, and the purified recombinant 6× His RALY expressed in *Escherichia coli*. As shown in Fig. 3D, the biotinylated IRES specifically interacted with the recombinant RALY. Furthermore, the association of cellular RALY with FMDV IRES was also verified in FMDV susceptible PK-15 and IBRS-2 cell lines (Fig. 3E and F), demonstrating that specific interaction between FMDV IRES and RALY is a general phenomenon in FMDV susceptible cells.

To further investigate the reciprocal interaction between RALY and FMDV IRES in FMDV-infected cells, BHK-21 lysates were immunoprecipitated with a specific anti-RALY antibody or with isotype anti-IgG at 5 hpi with FMDV (MOI = 1). The total RNAs extracted from these immunocomplexes were subjected to reverse transcription PCR (RT-PCR) using specific primers to FMDV IRES, FMDV 3D to 3′-UTR, or RPS16 and GAPDH (control primer). A specific DNA band was detected in the samples immunoprecipitated with RALY antibody but not with isotype IgG antibody and no antibody using specific primers to FMDV IRES, FMDV 3D to 3′-UTR. However, no RPS16 and GAPDH bands were detected in the precipitated samples, indicating that RALY interacts with the FMDV RNA during viral infection (Fig. 3G). Collectively, these results confirm that RALY directly interacts with FMDV IRES.

### RALY, through its RRM domain, interacts with D3 of FMDV IRES

**Fig 4:**
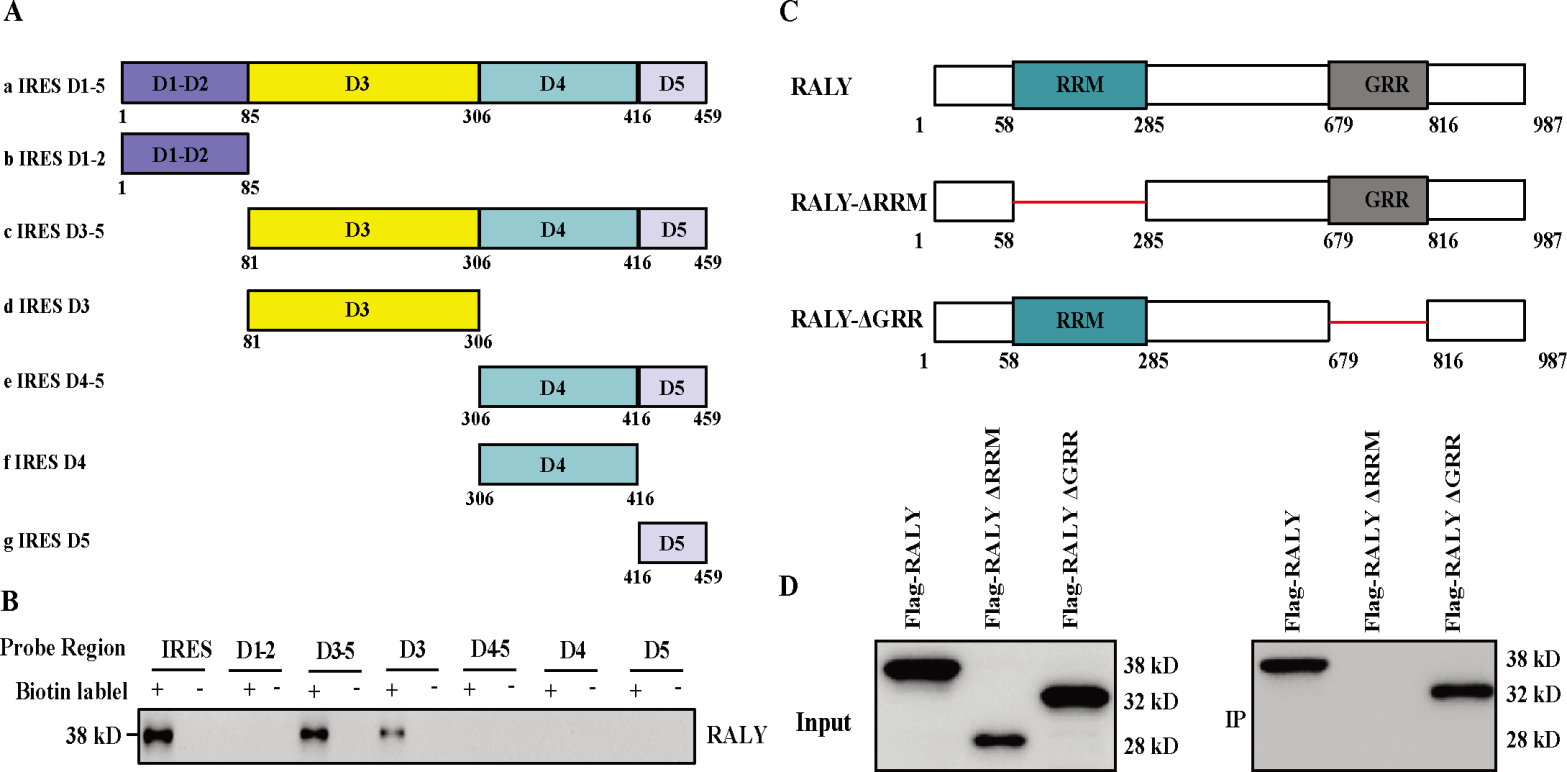
RALY, through its RRM, interacts with D3 of FMDV IRES. (**A**) Schematic representations of FMDV IRES truncations. Seven forms of IRES were generated: IRES D1-5, IRES D1-2, IRES D3-5, IRES D3, IRES D4-5, IRES D4, and IRES D5. (**B**) The full-length IRES and its truncated forms were transcribed *in vitro* and biotinylated. BHK-21 cell lysates were used to perform RNA pulldown assay with biotinylated IRES and its truncations. (**C**) Schematic diagram of RALY and its truncated mutants RALY-ΔRRM and RALY-ΔGRR. (**D**) Identification of interaction between RALY truncations and FMDV IRES. BHK-21 cells transfected with RALY or its truncated forms were collected at 24 h post-transfection and then pulled down with biotinylated FMDV IRES. The bound complexes were subjected to immunoblotting with anti-Flag antibody.

FMDV IRES belongs to type II IRES and has five important domains: D1–D5 (9). To confirm the domain(s) responsible for interacting with RALY, we designed six FMDV IRES truncated forms: D1-2, D3, D3–5, D4–5, D4, and D5 (Fig. 4A). The full-length IRES and its six truncated forms were biotinylated and performed RNA pulldown assay. These results show that RALY only specifically binds with full-length IRES and truncated forms D3-5, D3, indicating that the D3 region of FMDV IRES is responsible for the binding of RALY (Fig. 4B). RALY belongs to the hnRNP family and contains two important function domains: one RNA-binding motif (RRM) and one glycine-rich region (GRR). The main function of RRM is to recognize RNA, and the GRR domain may play an auxiliary role (31). According to the structural characteristics of RALY, we constructed two RALY truncated mutant forms, RALY-ΔRRM (without RRM domain) and RALY-ΔGRR (without GRR domain), to identify the domain(s) involved in the association with FMDV IRES by RNA affinity assay (Fig. 4C). We found that full-length RALY and truncated mutant forms RALY-ΔGRR can interact with FMDV IRES, but truncated mutant forms RALY-ΔRRM could not interact with FMDV IRES, demonstrating that RALY through its RRM domain interacts with FMDV IRES (Fig. 4D). In summary, we demonstrated that RALY through its RRM domain interacts with D3 of FMDV IRES.

### RALY inhibits the FMDV IRES-driven translation

**Fig 5:**
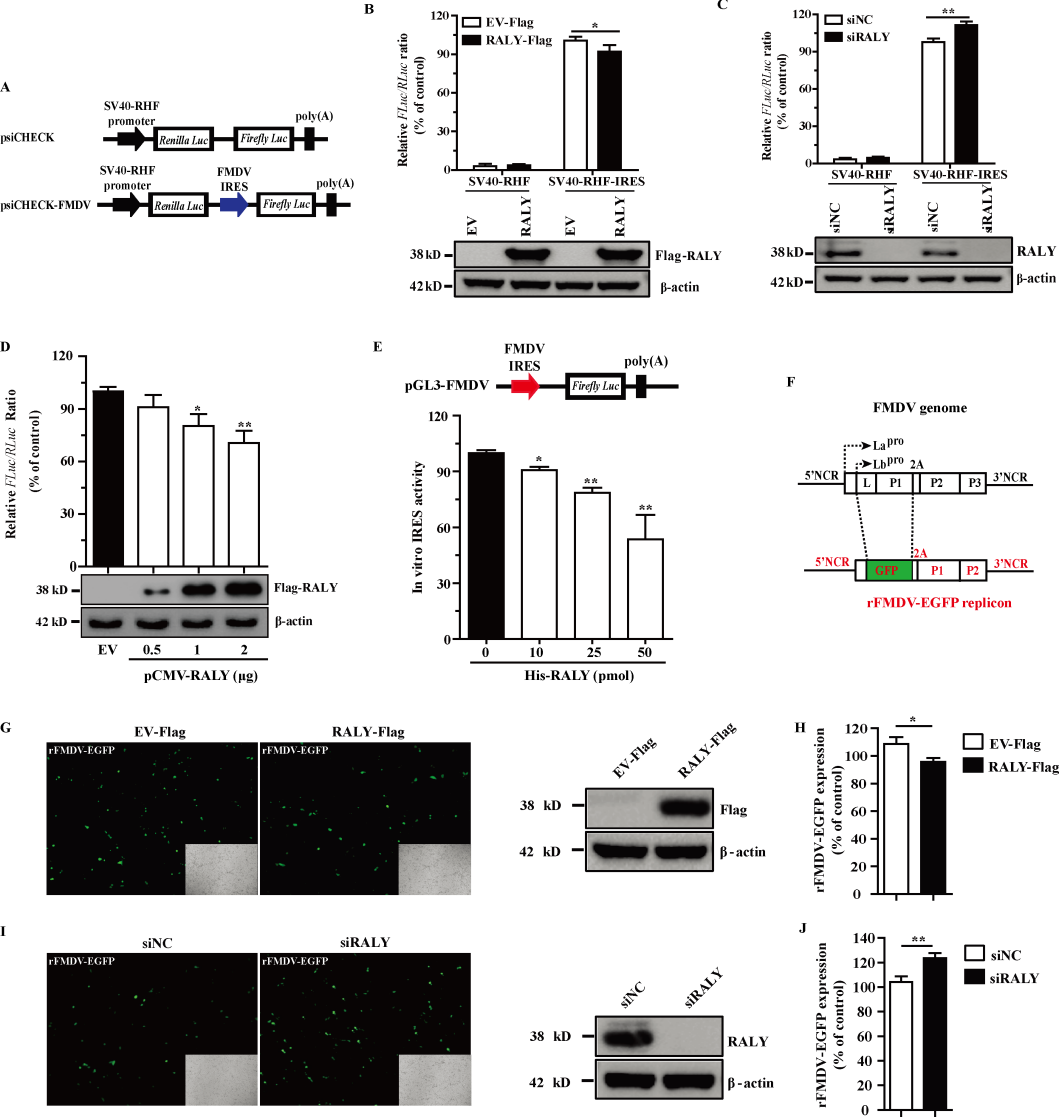
RALY inhibits the IRES-driven translation of FMDV. (**A**) Schematic illustration of bicistronic luciferase reporter plasmids. (**B and C**) The effect of RALY on FMDV IRES activity. RALY depletion or overexpression of BHK-21 cells was transfected with the luciferase reporter plasmids psiCHECK or psiCHECK-FMDV. At 24 hpt, the signal intensities of *Fluc* and *RLuc* were detected. RALY, Flag-RALY, and β-actin were analyzed by immunoblotting. (**D**) BHK-21 cells were transfected with different concentrations of RALY expression plasmids and psiCHECK-FMDV. Cell lysates were collected at 24 hpt, and the signal intensities of *Fluc* and *RLuc* were detected. (**E**) The RNA fragment with FMDV IRES and the *Fluc* coding sequence was incubated with RALY or BSA in RRL at 30°C for 40 min. The luciferase activity was determined with Steady-Glo. (**F**) Schematic diagram of the sub-genomic rFMDV-EGFP replicon. (G–J) BSR-T7 cells treated with the Flag-RALY plasmid or siRALY were transfected with the replicon rFMDV-EGFP. At 24 h post-transfection, the cells were subjected to fluorescence analysis by fluorescence microscope and flow cytometry. Statistical significance was assessed based on the *P* value. **P < 0.01.

The above research has proven that RALY directly interacts with FMDV IRES. To investigate the biological role of RALY binding to the FMDV IRES, BHK-21 cells were transfected with the bicistronic luciferase reporter plasmid psiCHECK-FMDV, which indicates a cap-dependent translation of the *RLuc* gene and an FMDV IRES-dependent translation of the *Fluc* gene (Fig. 5A). The relative activity was measured using a dual-luciferase assay to calculate the *RLuc/Fluc* ratio (Table 2). The result showed that the FMDV IRES activity was significantly decreased in RALY-overexpressing cells compared to the expression of empty vector (EV-Flag) (Fig. 5B), whereas an obvious increase of its activity was detected in the RALY-deficient cells (Fig. 5C). Moreover, a dose-dependent inhibitory effect of RALY overexpression on the FMDV IRES-dependent translation compared with cap-mediated translation was observed (Fig. 5D). The inhibition of RALY on FMDV IRES-dependent translation was further confirmed by cell-free translation system with a monocistronic construct composed of FMDV IRES and the *Fluc* gene. In this assay, the presence of RALY significantly inhibited FMDV IRES-dependent translation in a dose-dependent manner (Fig. 5E). Therefore, our results indicate that RALY has an inhibitory effect on FMDV IRES-driven translation in both the monocistronic and bicistronic luciferase reporter assays.

**TABLE 2:** *Fluc* and *RLuc* values corresponding to Fig. 5

*Fluc/RLuc*	EV-Flag	RALY-Flag	siNC	siRALY
SV40-RHF	18,567/2,576,570	26,268/2,578,260	11,524/1,448,720	20,807/1,398,733
	27,017/2,589,701	42,198/2,617,346	18,001/1,438,901	18,160/1,502,182
	22,972/2,501,592	24,805/2,588,059	22,038/1,460,541	26,602/1,424,433
SV40-RHF-IRES	862,531/2,599,413	778,985/2,615,648	472,263/1,436,460	511,878/1,419,890
	831,447/2,534,062	786,878/2,595,724	442,910/1,402,520	526,486/1,424,067
	890,642/2,568,357	745,275/2,605,091	475,437/1,424,378	505,474/1,441,533
*Fluc/RLuc*	EV	RALY 0.5 μg	RALY μg	RALY 2 μg
	20,675/109,698	19,613/118,632	18,703/112,855	16,365/108,688
	22,159/111,415	20,361/107,643	17,153/122,371	14,705/102,165
	22,961/118,379	18,916/113,217	16,729/104,265	12,377/107,197
*Fluc/RLuc*	His-RALY 0 pmol	His-RALY 10 pmol	His-RALY 25 pmol	His-RALY 50 pmol
	225,555/16,795,158	215,872/17,736,328	192,383/17,865,284	150,528/16,281,036
	239,739/17,519,516	207,129/17,164,482	200,524/19,788,322	105,296/17,687,355
	234,802/17,661,318	219,437/17,571,978	183,501/16,951,022	116,673/18,007,600

To further determine the effects of RALY on FMDV translation, a sub-genomic replicon of FMDV containing the EGFP reporter gene, rFMDV-EGFP, was constructed (Fig. 5F) and transfected into RALY-overexpressed or -depleted BSR-T7 cells (Fig. 5G and I). Consistent with the results in Fig. 5B through E, the flow cytometry analysis revealed that the specific fluorescence expression was reduced in RALY-overexpressing cells (Fig. 5G and H). On the contrary, replicon activity was enhanced when RALY was depleted (Fig. 5I and J). These data indicated that RALY represses the IRES-dependent translation of FMDV.

### FMDV 3C^pro^ antagonizes RALY-mediated repression via the proteasome pathway

**Fig 6:**
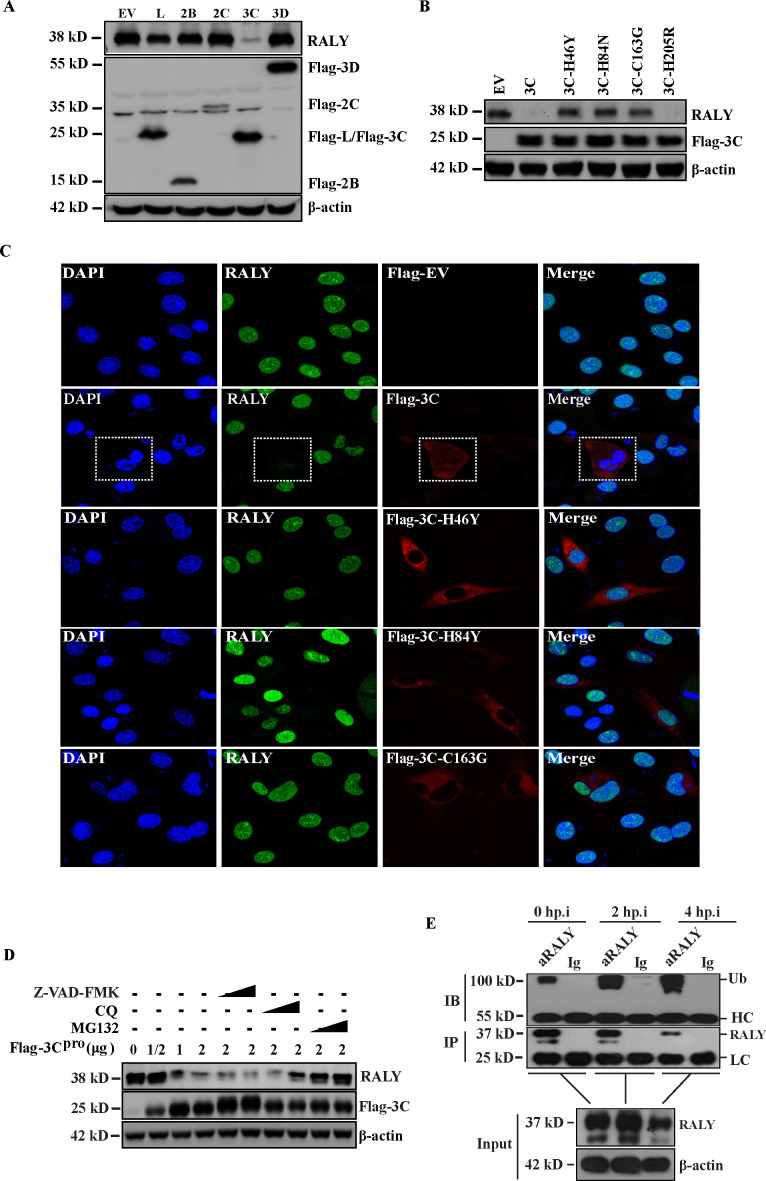
FMDV 3C^pro^ antagonizes RALY-mediated repression via the proteasome pathway. (**A**) BHK-21 cells were transfected with empty vector (EV), L^pro^, −2B, −2C, −3C^pro^, and −3D. Cell lysates were harvested and subjected to Western blot analysis. (**B**) BHK-21 cells were transfected with various mutants of Flag-3C^pro^, among which mutants 3C^pro^-H46Y, 3C^pro^-H84N, and 3C^pro^-163G lost proteinase activity, while H205R retained enzyme activity. (**C**) Flag-3C^pro^ and its plasmid mutants 3C^pro^-H46Y, 3C^pro^-H84N, and 3C^pro^-163G of FMDV were transfected into BHK-21 cells. Cells were fixed with 4% paraformaldehyde at 24 h post-transfection. immunofluorescence was also performed using primary anti-Flag and anti-RALY and then with secondary antibodies conjugated with TRITC (red) and FITC (green). (**D**) BHK-21 cells transfected with different amounts of Flag-3C^pro^ were treated with MG132 (two or 20 mM), Z-VAD-FMK (10 or 50 mM), or CQ (20 or 50 mM). After 24 h, the relative protein level of RALY was determined by Western blotting assay. (E) BHK-21 cells were infected with FMDV, and immunoprecipitation assay using an anti-RALY monoclonal antibody was performed on cell lysates. The polyubiquitylated RALY was determined by Western blotting with an anti-ubiquitin antibody.

Our results indicated that FMDV infection reduces the expression of RALY (Fig. 1). To further clarify how FMDV degrades RALY, non-structural protein plasmids of FMDV (Flag-L^pro^, −2B, −2C, −3C^pro^, and −3D) were transfected into BHK-21 cells. The results indicated that the ectopic expression of FMDV 3C^pro^ results in the degradation of RALY compared to the EV-Flag group, whereas the expression of other viral proteins did not (Fig. 6A). Surprisingly, no cleavage products were found in BHK-21 cells transfected with FMDV 3C^pro^. To determine whether the protease activity is required for the degradation of RALY by 3C^pro^, several previous constructs of mutant 3C^pro^ were transfected into BHK-21 cells (14). Western blot analysis showed that mutations 3C^pro^-H46Y, -D84N, and -C163G, which abolished its catalytic activity, did not result in the degradation of RALY. In contrast, the wild-type 3C^pro^ and mutant -H205R with enzyme activity resulted in the degradation of RALY (Fig. 6B). Meanwhile, immunofluorescence assay was also performed to confirm whether FMDV-3C^pro^ was responsible for the degradation of RALY. As shown in Fig. 6C, RALY was significantly degraded in BHK-21 cells transfected with FMDV 3C^pro^ compared with other mutant 3C^pro^. To reveal which protein degradation pathway was involved in FMDV 3C^pro^-mediated degradation of RALY, the caspase inhibitor Z-VAD-FMK, the lysosome inhibitor CQ, and the ubiquitin-proteasome inhibitor MG-132 were added into BHK-21 cells ectopically expressing FMDV 3C^pro^. Western blot results showed that RALY protein was restored using MG-132 (Fig. 6D). We also performed an immunoprecipitation assay to investigate the ubiquitin-proteasome degradation of RALY with an anti-RALY monoclonal antibody and then probed with the anti-ubiquitin antibody at 2 and 4 hpi. As shown in Fig. 6E, the RALY polyubiquitylation in BHK-21 cell-infected FMDV was enhanced following viral infection, indicating that FMDV 3C^pro^ degraded RALY protein. These results showed that 3C^pro^ antagonizes the repression of RALY for FMDV IRES-mediated translation and early virus assembly intermediate formation via the ubiquitin-proteasome pathway.

### RALY associates with translation initiation complex on FMDV IRES

**Fig 7:**
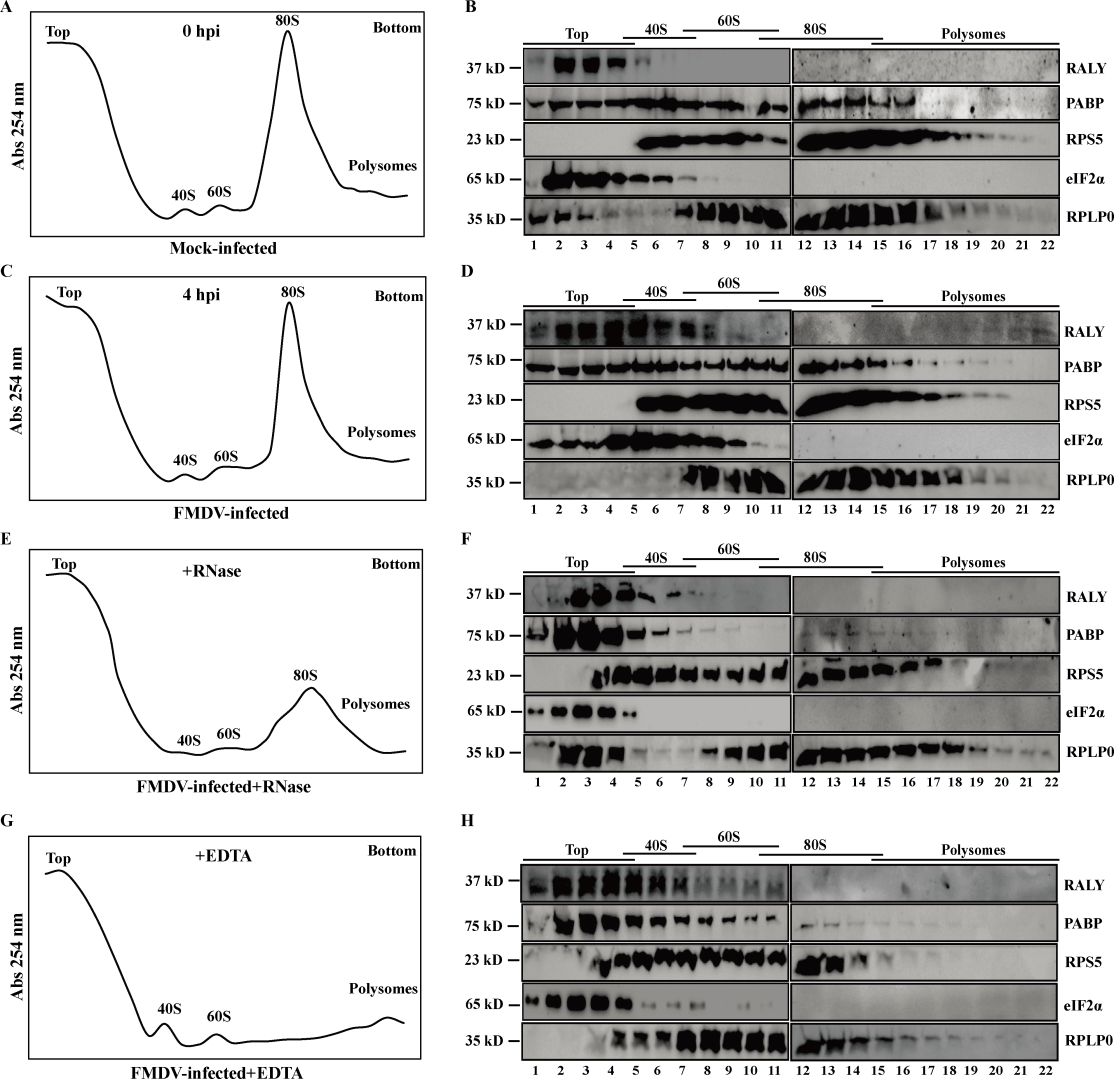
RALY associates with translation initiation complex on FMDV IRES. BHK-21 cells were treated with 0.1 mg/mL of cycloheximide for 15 min to arrest polysomes. Extracts from mock-infected (**A and B**) or FMDV-infected cells at 4 hpi (C–H) were collected and then treated with RNase I (**E and F**) or EDTA (**G and H**). The extracts’ supernatant was sedimented through 5%–50% sucrose density gradients to fractionate a subset of polysomes. Polysome profiles were generated by measuring the UV absorbance of each fractionation at 254 nm in each gradient fraction. The first peak contains free cytosolic light components (messenger ribonucleoprotein particles). The following peaks include ribosomal sub-units (40S and 60S) and translating monosomes (80S) (left). The remaining peaks of the profile represent polysomes. Collected fractions were also subjected to Western blotting to detect the dynamic changes of RALY and ribosomal sub-unit protein.

Previous research has proven that RALY mainly localized in the nucleus and partially in the cytoplasm. Meanwhile, we also demonstrated that RALY combines with FMDV IRES to inhibit viral translation in the cytoplasm (Fig. 1 and 3). Thus, these results prompted us to investigate the possible relationship between RALY and the cellular translation machinery by studying the co-sedimentation profile of RALY with ribosomal sub-units. After CHX pre-treatment, the extracts of mock- or FMDV-infected cells were harvested and ultracentrifuged on a 5%–50% (wt/vol) linear sucrose density gradients to fractionate the messenger ribonucleoprotein particles, ribosomal sub-units (40S and 60S), monosomes (80S), and polysomes. As shown in Fig. 7A and C, the ribosome profiles were recorded by measuring the UV absorbance of each fractionation at 254 nm (OD_254_) and visualizing the distribution of ribosomal proteins using Western blotting (Fig. 7B and D). The component of 40S RPS5 and 60S ribosomal protein P0 (RPLP0) were used as a positive control of 40S and 60S ribosome sub-units. The poly A-binding protein (PABP) was distributed in almost all gradients and used as a negative control. The protein translation initiation factor eIF2α was mostly fractionated at the top of the gradient and partially sedimented in the 40S and 60S fractions. Compared with the mock-infected group, more RALY appeared in the 40S gradient fractions in FMDV-infected cells, suggesting that RALY may associate with 40S initiating complexes dependent on FMDV IRES.

To determine if the association of RALY with 40S initiating complexes was RNA-dependent, cell lysates were treated with RNase I before fractionation. As shown in Fig. 7E, the treatment with RNase I degraded single-stranded RNA and induced the disassembly of the vast majority of the 80S and polysomes. The sedimentation profile of RALY was primarily distributed to the low-density of sucrose gradient, similar to PABP and eIF2α (Fig. 7F), suggesting that RALY may be related to the formation of FMDV IRES-dependent translation initiation complexes. To further validate the association of RALY with the 40S sub-unit fractions, the cell lysate was treated with EDTA before sucrose fractionations. This treatment destroyed the interaction between the 40S and 60S sub-units of the ribosome. As shown in Fig. 7G, the polysomes, and 80S peaks disappeared, while 40S and 60S sub-units remained. Under these conditions, the sedimentation profile of RALY did not change compared with the FMDV-infected group (Fig. 7D), while RPS5 and RPLP0 were significantly reduced in the 80S and polysome fractions (Fig. 7H). These results indicate that RALY is bound to the 40S initiating complexes and may participate in the assembly of translation initiation complexes on FMDV IRES.

### RALY blocks 80S ribosome assembly on FMDV IRES

**Fig 8:**
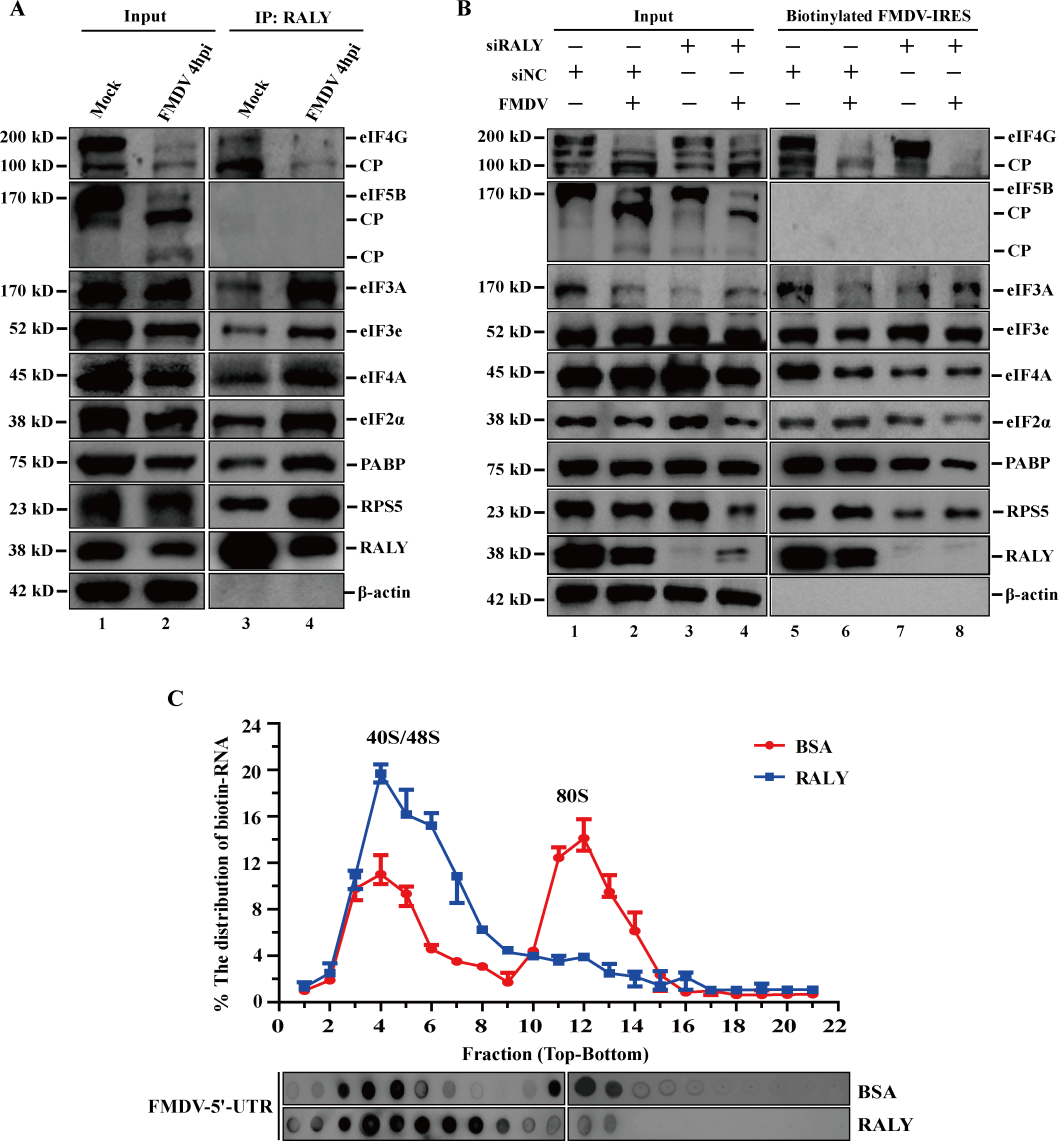
RALY blocks 80S ribosome assembly on FMDV IRES. (**A**) Extracts from mock- and FMDV-infected BHK-21 cells were subjected to immunoprecipitation assay at 4 hpi with an anti-RALY antibody (lanes 3 and 4). The proteins associated with translation initiation complexes were analyzed by Western blotting. (**B**) BHK-21 cells were transfected with siRALY and siNC. After 36 h, the cells were infected by FMDV. Cell lysates were harvested at 4 hpi and subjected to an RNA pulldown assay with biotinylated FMDV IRES. The proteins associated with translation initiation complexes were analyzed by Western blotting (lanes 5–8). (**C**) Biotin-RNA of FMDV-5′-UTR and recombinant RALY protein or BSA control were incubated in RPL at 30℃ for 40 min. The ribosome complexes were fractionated by sucrose density gradient ultracentrifugation. The distribution of biotin-labeled RNA was detected by dot blotting with streptavidin-HRP and analyzed by densitometry.

FMDV shuts down the host cellular cap-dependent translation process by cleaving the translation initiation factors eIF4G and eIF5B and recruiting ribosome and many eIFs (such as eIF2, eIF3, and eIF4) to translate its viral protein via FMDV IRES. To further investigate whether RALY participates in the translation initiation complexes through the IRES, an immunoprecipitation assay was performed to detect the participants associated with translation initiation factors using anti-RALY monoclonal antibodies from the extracts of mock- and FMDV-infected BHK cells. The Western blot analysis showed that RALY specifically co-precipitated with PABP, the 40S component RPS5 and the majority of translation initiation factors, including eIF4G (cleaved eIF4G), eIF3A, eIF3e, eIF4A, and eIF2α, but not with eIF5B (Fig. 8A). In addition, we found that the binding ability of RALY in the FMDV-infected group was stronger than that in the mock-infected group (Fig. 8A, lanes 3 and 4), suggesting RALY prefers to bind in FMDV IRES-dependent rather than cap-dependent translation initiation complex. These results indicate that RALY may participate in the FMDV IRES-mediated translation initiation complexes.

To further explore the specific function of RALY in FMDV IRES-mediated translation initiation complexes, we performed an RNA affinity assay using the biotinylated-IRES and extracts from mock- and FMDV-infected BHK-21 cells. As shown in Fig. 8B, the depletion of RALY did not significantly affect the expression levels of the indicated translation initiation factors (lanes 3 and 4). eIF4G (cleaved eIF4G), eIF3A, eIF3e, eIF4A, and eIF2α were enriched in FMDV IRES-binding protein complexes, but not eIF5B (lanes 5–8). However, the specific interaction did not change when RALY was knocked down, except for eIF4G (lanes 7 and 8). These observations suggest that RALY is associated with IRES-driven translation but does not directly participate in the assembly of translation initiation complexes.

Considering that RALY specifically binds to FMDV IRES-mediated translation initiation complex and reduces IRES activity, we speculate that it may affect the assembly of 80S ribosome by interfering with the recruitment of 60S ribosomes to 40S ribosomes. To clarify the mechanism of RALY inhibiting FMDV IRES activity, ribosome assembly was detected in the RRL system. The biotin-labeled FMDV IRES RNA and recombinant RALY protein or BSA control were incubated with RRL. The ribosome assembly mixtures were ultracentrifuged by sucrose density gradient to separate ribosomal complexes and detected by dot blotting. Consistent with previous reports, the formation of 80S ribosome was observed in the BSA control group after 40 min of incubation. In contrast, its peak was significantly reduced in the presence of recombinant RALY protein. Most of the biotinylated RNA was detained in the 40S/48S peak (Fig. 8C). Therefore, the inhibitory effect of RALY on FMDV IRES activity is due to the blocking of 80S ribosome assembly after binding with 40S ribosome.

## DISCUSSION

**Fig 9:**
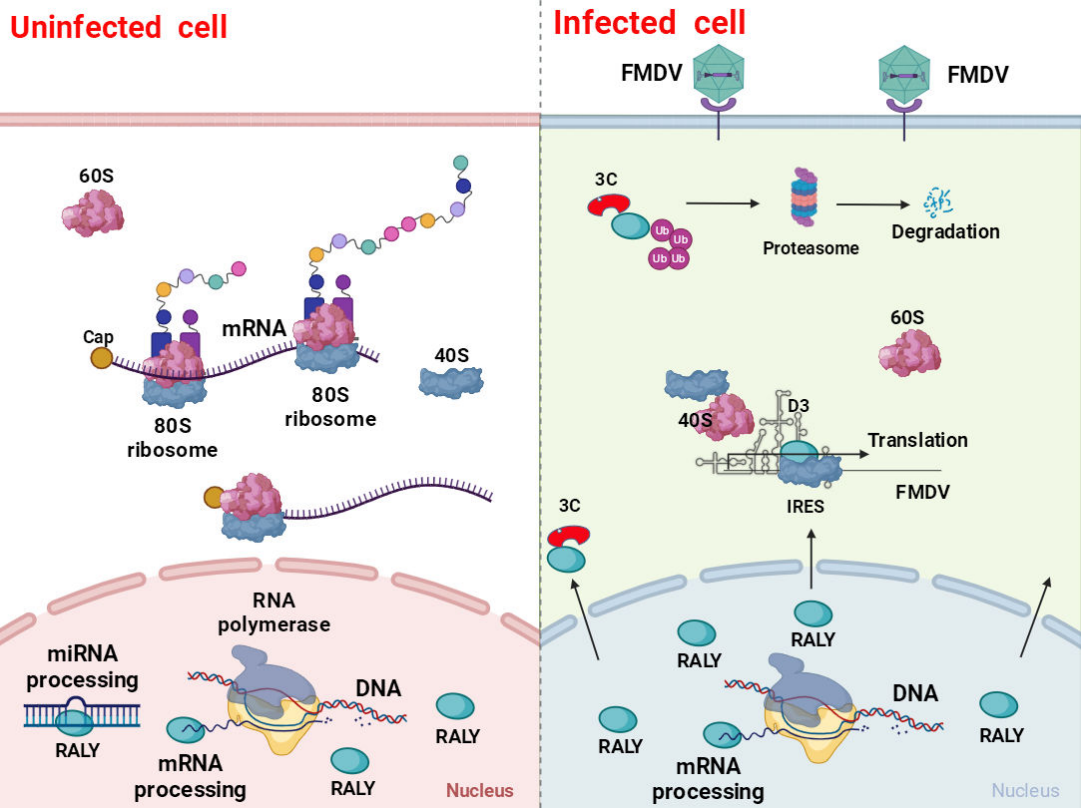
Schematic diagram of negative RALY regulation of FMDV infection. RALY is an RNA-binding protein mainly located in uninfected cells’ nucleus and involved in pre-mRNA or miRNA splicing and processing. However, RALY is transported to the cytoplasm from the nucleus with FMDV infection, where it binds to FMDV IRES-D3 and 40S initiating complexes to inhibit FMDV IRES-driven translation by blocking 80S ribosome assembly on FMDV IRES. Conversely, 3C^pro^ of FMDV antagonizes RALY-mediated inhibition by the ubiquitin-proteasome pathway.

Regulating IRES-dependent translation is a critical step for picornaviral propagation, which impacts virus virulence and pathogenicity. As an indispensable replication element, the IRES recruits a subset of translation initiation factors and various RNA-binding proteins to drive the translation of the viral proteins and regulate viral RNA synthesis (9). Thus, identifying specific host factors associated with the IRES-driven translation of picornavirus would help us better understand virus-host interactions and pathogenic mechanisms. In this study, we identified a novel RNA-binding protein, RALY, as a novel host restriction factor that downregulates FMDV replication by inhibiting IRES-driven translation (Fig. 9).

As an important member of the hnRNP family, RALY plays an important role in many processes of mRNA metabolism, such as mRNA splicing, stability, and the translational regulation of specific mRNAs in many different cell types, especially in cancer cells (32–34). In addition, it was reported that RALY significantly promotes the degradation of the PEDV N protein to restrict viral replication through a RALY-MARCH8-NDP52-autophagosome pathway (27). Here, we demonstrated that the interaction between RALY and FMDV IRES is a common phenomenon among different FMDV-susceptible cells (Fig. 3). Meanwhile, we also revealed that RALY interacts with the D3 of FMDV IRES via its RRM domain (Fig. 4), implying that RALY may influence FMDV IRES-driven translation. Further functional analysis indicated that RALY is a negative regulator of FMDV translation using dual-luciferase reporter assay and rFMDV-EGFP replicon (Fig. 5). These results indicated that RALY represses the FMDV IRES-driven translation. To initiate IRES-driven translation, the highly conserved virus IRES structure recruits some canonical initiation factors and 40S sub-unit with high affinity, subsequently assembling an 80S ribosome for the translation of viral proteins (9, 13). The 80S ribosome assembly on the IRES involves multiple steps manipulated by specific IRES structure domains. Interaction of the cricket paralysis virus intergenic region IRES with ribosomal protein S25 (eS25) is required to form a stable 40S-IRES translation complex (35). The deletion of IIId2 from the CSFV and BDV IRES elements impairs translation initiation by inhibiting the assembly of 80S ribosomes (36). hnRNP K directly binds to domains II, III, and IV of the FMDV IRES and inhibits IRES-mediated translation by interfering with the recognition of PTB and impairing the formation of the translation initiation complex (37). Based on the characteristics of ribosome assembly, we speculated that the inhibitory effect of RALY on FMDV IRES-mediated translation may repress the pre-initiation complex assembly or block the formation of the 80S ribosome complex after 40S/48S formation. Our results indicated that the depletion of RALY does not affect the assembly of translation initiation complexes but impairs 80S ribosome assembly by interfering with the recruitment of 60S ribosomes to 40S ribosomes (Fig. 8).

Since RALY is a multifunctional RNA-binding protein generally involved in the alternative splicing of mRNA and miRNA processing (32, 38), we further explore the effect of RALY on FMDV RNA stability via additional drug experiments. These results indicated that RALY was not involved in viral mRNA stability (Fig. 2G and H). Given the inhibitory effect of RALY on FMDV IRES-driven translation by blocking the formation of 80S ribosome, we analyzed the function of RALY in the switch between the translation and replication during the FMDV infection. Our results showed that RALY does not affect viral RNA synthesis (Fig. 2E and F). Based on the above results, we demonstrate that RALY is not required for maintaining FMDV RNA stability and negative-strand RNA synthesis.

FMDV has a short replication cycle in infected cells and promptly activates the host anti-viral system to suppress FMDV replication (39). To retain host adaption, viruses have evolved strategies to counteract infected host cells’ very sophisticated defense mechanisms (40). L^pro^ and 3C^pro^ of FMDV play the most important functions in inhibiting host protein synthesis and cleavage (41). Furthermore, some members of the hnRNP family, such as PABP, PTB, and hnRNP K, were cleaved by the L^pro^ and 3C^pro^ of FMDV to antagonize their inhibitory activity (16, 37). This study found that RALY inhibits FMDV IRES-driven translation by interrupting 60S ribosome recruitment. However, RALY was antagonized by the FMDV 3C^pro^ for the survival and propagation of the virus itself (Fig. 1 and 8). Meanwhile, we also discovered that EMCV, SVA, and other picornaviruses significantly degraded RALY protein (Fig. 1D and F), indicating that the degradation of RALY was not restricted to FMDV in picornaviruses (Fig. 1 and 6).

In conclusion, the work presented here revealed that RALY acts as a novel host restriction factor that binds to the FMDV IRES and downregulates FMDV IRES-driven translation, impairing the formation of 80S ribosome after binding with 40S ribosome assembly during FMDV infection. However, the obvious advantage of RALY for hindering FMDV propagation was antagonized by viral 3C^pro^ protease. Therefore, this study revealed a novel role for RALY in FMDV replication and contributed to a better understanding of the virus-host interactions and developing anti-viral strategies (Fig. 9).

## Data Availability

Data sharing is not applicable to this article as no new data were created or analyzed in this study.
